# Epidemiologic Associations Vary Between Tetracycline and Fluoroquinolone Resistant *Campylobacter jejuni* Infections

**DOI:** 10.3389/fpubh.2021.672473

**Published:** 2021-06-28

**Authors:** Jose A. Rodrigues, Wonhee Cha, Rebekah E. Mosci, Sanjana Mukherjee, Duane W. Newton, Paul Lephart, Hossein Salimnia, Walid Khalife, James T. Rudrik, Shannon D. Manning

**Affiliations:** ^1^Department of Microbiology and Molecular Genetics, Michigan State University, East Lansing, MI, United States; ^2^Department of Pathology, University of Michigan, Ann Arbor, MI, United States; ^3^School of Medicine, Wayne State University, Detroit, MI, United States; ^4^Sparrow Hospital, Lansing, MI, United States; ^5^Detroit Medical Center University Laboratories, Detroit, MI, United States; ^6^Michigan Department of Health and Human Services, Bureau of Laboratories, Lansing, MI, United States

**Keywords:** *Campylobacter*, antibiotic resistance, epidemiology, ciprofloxacin resistance, tetracycline resistance, risk factor

## Abstract

*Campylobacter jejuni* is the leading cause of bacterial gastroenteritis and antibiotic resistant *C. jejuni* are a serious threat to public health. Herein, we sought to evaluate trends in *C. jejuni* infections, quantify resistance frequencies, and identify epidemiological factors associated with infection. *Campylobacter jejuni* isolates (*n* = 214) were collected from patients via an active surveillance system at four metropolitan hospitals in Michigan between 2011 and 2014. The minimum inhibitory concentration for nine antibiotics was determined using microbroth dilution, while demographic and clinical data were used for the univariate and multivariate analyses. Over the 4-year period, a significant increase in the recovery of *C. jejuni* was observed (*p* ≤ 0.0001). Differences in infection rates were observed by hospital and several factors were linked to more severe disease. Patients residing in urban areas, for instance, were significantly more likely to be hospitalized than rural residents as were patients over 40 years of age and those self-identifying as non-White, highlighting potential disparities in disease outcomes. Among the 214 *C. jejuni* isolates, 135 (63.1%) were resistant to at least one antibiotic. Resistance was observed for all nine antibiotics tested yielding 11 distinct resistance phenotypes. Tetracycline resistance predominated (*n* = 120; 56.1%) followed by resistance to ciprofloxacin (*n* = 49; 22.9%), which increased from 15.6% in 2011 to 25.0% in 2014. Resistance to two antibiotic classes was observed in 38 (17.8%) isolates, while multidrug resistance, or resistance to three or more classes, was observed in four (1.9%). Notably, patients with ciprofloxacin resistant infections were more likely to report traveling in the past month (Odds Ratio (OR): 3.0; 95% confidence interval (CI): 1.37, 6.68) and international travel (OR: 9.8; 95% CI: 3.69, 26.09). Relative to patients with only tetracycline resistant infections, those with ciprofloxacin resistance were more likely to travel internationally, be hospitalized and have an infection during the fall or summer. Together, these findings show increasing rates of infection and resistance and highlight specific factors that impact both outcomes. Enhancing understanding of factors linked to *C. jejuni* resistance and more severe infections is critical for disease prevention, particularly since many clinical laboratories have switched to the use of culture-independent tests for the detection of *Campylobacter*.

## Introduction

*Campylobacter* spp. are a leading cause of bacterial gastroenteritis infections worldwide (1) and represent the most common cause of foodborne infections in the U.S. since 2013 (2). While *C. jejuni* causes a vast majority of human infections, other species including *C. coli, C. upsaliensis, C. lari, C. fetus, C. insulaeingrae*, and *C. hyointestinalis*, are also important (3). Collectively, these pathogens were estimated to cause 1.5 million infections in the U.S. each year, contributing to 13,000 hospitalizations and 120 deaths (4). In 2018, the Centers for Disease Control and Prevention (CDC) estimated the incidence of campylobacteriosis to be 19.6 cases per 100,000 individuals, which had increased from 12.0 cases per 100,000 in 2015–2017, among sites participating in the Foodborne Diseases Active Surveillance Network (FoodNet) (2).

Clinical manifestations of campylobacteriosis include fever, abdominal pain, vomiting, weight loss, chills, fatigue, myalgia, malaise, and acute watery or bloody diarrhea (1). The incubation period is typically 1–4 days after exposure, and the severity of symptoms tends to vary by bacterial density and strain (5). Post-infectious immune sequalae such as Guillain-Barré Syndrome, Miller-Fisher syndrome, and reactive arthritis, have been linked to *Campylobacter* infection as have inflammatory bowel disease, esophageal and colo-rectal cancers, and extra-intestinal infections like bacteremia and meningitis (6). Although most infections are self-limiting, antibiotics are often needed for immunocompromised patients or those with more severe or persistent infections (7).

Water, poultry and livestock are common reservoirs for *C. jejuni* (8). Transmission to humans typically occurs via consumption of contaminated food products, and direct contact with animal or environmental reservoirs (9). According to a meta-analysis of 72 studies, the key risk factor for campylobacteriosis was international travel, yet consumption of undercooked chicken and direct exposure to *Campylobacter* from the environment or farm animals were also important (10). Regardless, it is important to note that risk factors often vary by geographic location even across the U.S., with different FoodNet sites reporting considerable variation in the frequency of infections (11). In addition, the FoodNet sites were not selected to be representative of the U.S. population and were shown to have an unequal representation of all racial and ethnic groups and contained fewer individuals living below the poverty level (12, 13).

*Campylobacter jejuni* has also been designated a serious antibiotic resistant threat resulting in 448,400 resistant infections and 70 deaths each year (14). Resistance to ciprofloxacin, a fluoroquinolone used to treat more severe human infections, increased in the U.S. from 13% in 1997 to 25.3% in 2015 (14, 15). *Campylobacter jejuni* resistance to multiple drug classes has also increased over time (16) and resistant isolates have been linked to more severe infections requiring lengthier hospitalizations (17). Because NARMS does not utilize data from each state and the Midwest region only receives a subset of *Campylobacter* isolates from the Minnesota FoodNet site for testing (18), these resistance frequencies and trends may not be representative of those in other locations. Additionally, many clinical laboratories have shifted to the use of culture-independent tests to detect *Campylobacter* infections, which can obscure actual rates of resistance circulating within patient populations and prevent the identification of risk factors for resistant infections. Indeed, a 2019 FoodNet report noted that 42% of *Campylobacter* infections were detected using a culture-independent test (2). This shift is concerning and highlights the need for more culture-based studies to better define the epidemiology of and resistance phenotypes in this common foodborne pathogen.

Herein, we sought to describe the susceptibility profiles for 214 *C. jejuni* isolates cultured from patients with campylobacteriosis during surveillance activities in Michigan (2011–2014) and to identify risk factors for both susceptible and resistant infections. We also sought to make comparisons to national data available through NARMS since *Campylobacter* resistance is not monitored in Michigan via NARMS (19). Studies such as these highlight the importance of using culture-based diagnostic tests to more accurately monitor resistance phenotypes and frequencies in distinct geographic locations to identify potential exposures and risk factors that may be state and/or region specific.

## Materials and Methods

### Strain Source and Speciation

*Campylobacter* isolates were recovered from stools of patients with campylobacteriosis between 2011 and 2014 via an active surveillance system at four metropolitan hospitals located in Detroit, Grand Rapids, Ann Arbor, and Lansing, Michigan. Isolates were transported to the Michigan Department of Health and Human Services (MDHHS) and stored in 10% skim milk at −80°C until use.

Isolates were thawed and cultured on Tryptone Soy Agar (TSA) containing 5% sheep blood and cefoperazone (20 μg), amphotericin B (4 μg/mL), and vancomycin (20 μg/mL) in microaerophilic conditions (20). DNA was extracted and multiplex PCR was performed to classify the species of each *Campylobacter* isolate using a previously described protocol (21). Briefly, the Kapa2G Taq (Kapa Biosystems; Wilmington, MA) was used for PCR amplification using the following conditions: denaturation at 95°C for 15 min followed by 25 cycles of 95°C for 30 s and 58°C for 1 min and 30 s and 72°C for 8 min. Roughly 91 of the 214 (43%) *C. jejuni* isolates included in the analysis were characterized previously (22).

### Antimicrobial Susceptibility Profiling

The minimum inhibitory concentration (MIC) was determined for nine antibiotics using microbroth dilution utilizing Sensititre^TM^ Campylobacter Campy AST plates (ThermoFisher; Waltham, MA) according to the manufacturer's protocols. The antibiotics (classes) were: ciprofloxacin (fluoroquinolone), nalidixic acid (quinolone), azithromycin (macrolide), erythromycin (macrolide), tetracycline, florfenicol (phenicol), telithromycin (ketolide), clindamycin (lincomycin), and gentamicin (aminoglycoside). *Campylobacter jejuni* ATCC 33560 was used a control. The European Committee on Antimicrobial Susceptibility Testing (EUCAST) standards were used, as per the current NARMS protocol, for classifying isolates as resistant or susceptible (19). NARMS data were extracted from isolates collected in the same time period for comparison. Isolates with any ciprofloxacin resistance and any tetracycline resistance were counted; these two categories were not mutually exclusive as some isolates had resistance to both drugs. A subset of data submitted from NARMS Region 5 representing the Midwest (Ohio, Indiana, Michigan, Illinois, Wisconsin, and Minnesota and 34 federally recognized tribes) were also included in this analysis for comparison.

### Epidemiological Variables and Data Analysis

Demographics, exposures, and clinical data were extracted from the Michigan Disease Surveillance System (MDSS), an online database containing epidemiological data for notifiable infections. The sample collection date was used to classify the season as follows: spring (March, April, May), summer (June, July August), fall (September, October, and November), and winter (December, January, and February). Cases reporting a history of travel in the past month were classified as traveling domestically (within the U.S.) or internationally. Michigan counties were designated as urban or rural based on data presented in a National Center for Health Statistics report (23); all but 10 Michigan counties were considered rural. Cattle densities per county were obtained from a 2019 report (24), and the high vs. low categories were developed based on the average number of cattle in all Michigan herds with data available.

Chi-square tests were used for dichotomous variables to identify associations between the dependent and independent variables, while the Mantel-Haenszel Chi-square test was used to examine trends. Differences in proportions were evaluated using the Chi-square test for equal proportions and for variables with small sample sizes, or less than five per cell, the Fisher's exact test was used. A *p* ≤ 0.05 was considered significant for each test, however, all variables yielding a *p* ≤ 0.20 in the univariate analysis were included in the multivariate analyses. Potential confounders such as age, sex, and residence location, were also included in the forward logistic regression analyses to identify predictors of each outcome. Odds ratios (ORs) and their 95% confidence intervals (CIs) were calculated to describe the magnitude of each association. SAS version 9.4 (SAS Institute, Cary, NC, USA) and Epi Info™ version 7 were used.

## Results

### Recovery of *Campylobacter* in Michigan

In all, 277 *Campylobacter* isolates were recovered from Michigan residents diagnosed with campylobacteriosis at four large metropolitan hospitals between January 2011 and December 2014. Approximately 234 (84.5%) of the isolates were viable and could be speciated using PCR. Among these, 217 (92.7%) were classified as *C. jejuni*, while 15 (6.0%) were *C. coli*; two isolates (0.9%) were characterized as *C. upsaliensis*. Given that *C. jejuni* was the most common species, the analysis was restricted to these isolates and cases. Three additional isolates from residents living outside of Michigan were also excluded from the analysis.

Significant variation in the recovery of *C. jejuni* was observed across hospitals (*p* ≤ 0.0001), with most isolates (*n* = 174; 82.1%) coming from two sites; the hospital location was missing for three isolates. The frequency of *C. jejuni* at each site was 42.9% (*n* = 91), 39.2% (*n* = 83), 7.6% (*n* = 16), and 9.9% (*n* = 21). A significant difference in the recovery of *C. jejuni* isolates was also observed over time with 57.5% (*n* = 123) of the infections occurring in 2013 and 2014 (*p* ≤ 0.0001). Differences were also observed by season since more isolates were recovered in the summer and fall months (*n* = 158; 73.8%) compared to the winter and spring (*n* = 56; 26.2%) (*p* ≤ 0.0001). Moreover, a greater proportion of cases resided in urban (*n* = 119; 62.0%) vs. rural (*n* = 73; 38.0%) areas (*p* = 0.0009).

### Demographics and Exposure History of *C. jejuni* Cases

Among the 214 *C. jejuni* cases from Michigan residents, 110 (54.4%) were male and 53.1% (*n* = 113) were between the age of 19 and 65 years; the age was missing for one case (Supplementary Table 1). Sixty-five (30.5%) cases represented children between 1 day and 9 years of age. Twenty (30.8%) of these children were ≤1 year old and 33 (50.8%) were between 1 and 5 years of age. Among the 17 elderly patients over 65 years, over half (*n* = 11) were between 70 and 87 years of age. Significantly more cases self-identified as White/Caucasian (*n* = 137; 79.7%), though a subset self-identified as Black/African American (*n* = 17; 9.8%), Asian (*n* = 6; 3.5%), or another race (*n* = 13; 7.5%). Thirteen (8.8%) cases self-identified as Hispanic/Latino and 25 (19.1%) self-identified as Arab, however, up to 83 (38.8%) cases did not indicate their ethnicity.

The majority (*n* = 88; 59.9%) of cases did not travel in the month prior to infection compared to 40.9% of cases who did. Among 59 of the 61 cases reporting their travel location, 18.4% (*n* = 27) traveled internationally and 22.4% (*n* = 33) reported domestic travel. While more of these cases traveled during the summer (*n* = 30; 44.4%) and fall (*n* = 18; 29.5%) as opposed to the winter and spring (*n* = 13; 21.3%), the difference was not significant (*p* = 0.26).

Despite the greater proportion of cases reporting animal contact prior to illness onset (*n* = 96; 64.0%), multiple animal species were reported. Among these cases, 88 (91.7%) cases reported contact with domestic animals, 13 (13.5%) with livestock and 11 (11.5%) with birds or poultry. Seventeen cases (17.7%) reported contact with other animals and three (3.1%) had contact with reptiles. Furthermore, most (*n* = 199; 81.5%) cases drank municipal and/or bottled water and consumed poultry (*n* = 115; 87.8%) up to a week prior to symptom onset. Food history data, however, was not available for up to 38.8% of the cases.

Because significantly more cases lived in urban areas, we also sought to determine whether any factors were associated with urban residence (Supplementary Table 2). Importantly, the odds of hospitalization for urban residents was significantly greater (*n* = 34; 73.9%) than rural residents (*n* = 12; 26.1%), yet no differences in symptoms were observed. Urban patients were also significantly less likely to report any travel in the past month, either domestic or international, and were less likely to be between 19 and 40 years of age than rural patients. Indeed, 83.6% (*n* = 46) of children < 10 years of age resided in an urban area compared to 16.4% (*n* = 9) for rural children. Although exposure to livestock and Arab ethnicity were significantly associated with rural and urban residence, respectively, the sample sizes were small for each variable and many records had missing data.

### Clinical Symptoms and Association with More Severe Infections

Among the subset of cases reporting symptoms, diarrhea (95.0%) was the most common followed by abdominal pain (69.7%), nausea (41.3%), and fatigue (40.9%). Only 36.2% of cases reported the presence of bloody diarrhea, while 34.5, 28.7, and 26.6% reported chills, body aches, and headaches, respectively. In all, 46 (25.3%) patients were hospitalized ranging from 1–11 days with an average of 3 days.

In addition to urban residence, several other factors were associated with hospitalization, a marker for more severe disease, in the univariate analysis (Supplementary Table 3). An increasing odds of hospitalization was observed as age increased. Compared to adults between years, adult patients between 41 and 65 years and the elderly over 65 years were significantly more likely to be hospitalized. The same was true when children <9 years was used as the reference group. Patients self-reporting nausea and fatigue were also more likely to be hospitalized as were patients self-identifying as non-White. By contrast, patients reporting domestic or international travel in the month prior to symptom onset were significantly less likely to be hospitalized. No association was observed for sex, season, source of drinking water, or any other symptoms, and no differences were detected when the analysis was limited to only those individuals without a recent history of international travel.

Controlling for potential confounders such as residence type (urban vs. rural), sex, season, and age, multinomial logistic regression identified the oldest age groups, 41–65 years (adjusted OR: 6.1; 95 CI: 2.37, 15.70) and >65 years (adjusted OR: 10.5; 95 CI: 2.63, 42.19), to be predictors of hospitalization relative to the younger age groups. International travel in the past month (adjusted OR: 0.3; 95% CI: 0.07,0.94), non-White race (adjusted OR: 4.8; 95% CI: 1.62, 14.01), and nausea (adjusted OR: 2.8; 95% CI: 1.15, 6.68) were also independently associated with hospitalization.

### Antibiotic Resistance Phenotypes and Frequencies

**Figure 1:**
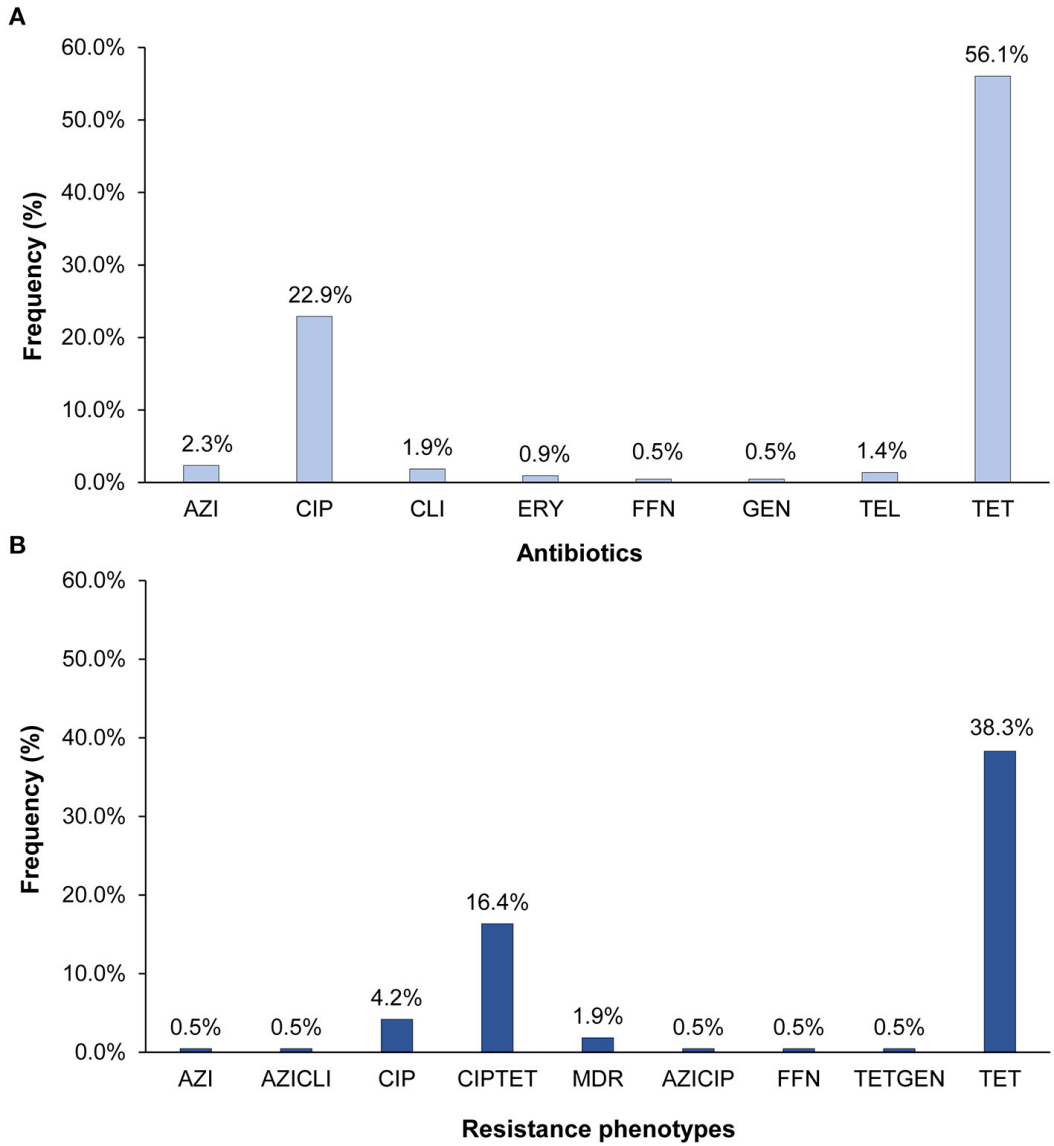
Percentage of the 214 *Campylobacter jejuni* isolates with **(A)** resistance to eight different antibiotics and **(B)** distinct antibiotic resistance phenotypes. AZI, azithromycin; CIP, ciprofloxacin; CLI, clarithromycin; ERY, erythromycin; FFN, phenicol; GEN, gentamicin; TEL, telithromycin; TET, tetracycline. The multidrug resistant (MDR) phenotype includes isolates with resistance to CIPAZIERYCLITEL, CIPTETCLI, and CIPTETTEL. All CIP resistant isolates were also resistant to nalidixic acid.

Resistance was detected in 63.1% (*n* = 135) of the 214 *C. jejuni* isolates and at least one isolate was resistant to each of the nine antibiotics tested. Tetracycline resistance (*n* = 120; 56.1%) predominated followed by resistance to ciprofloxacin (*n* = 49; 22.9%) (Figure 1A). Fewer than five isolates had resistance to clindamycin, azithromycin, and telithromycin, and only one isolate was resistant to gentamicin and another to phenicol. All isolates with ciprofloxacin resistance were also resistant to nalidixic acid. Among the 135 resistant isolates, 93 (43.5%) were resistant to one class of antibiotics, whereas 38 (17.8%) were resistant to two. In all, 11 different *C. jejuni* resistance phenotypes that varied in frequency (Figure 1B). Five of these phenotypes included tetracycline resistance, six included ciprofloxacin resistance, and three phenotypes included both. The predominant phenotypes were tetracycline resistance alone (*n* = 82; 38.3%) and in combination with ciprofloxacin (*n* = 35; 16.4%). Multidrug resistance (MDR), which is defined as resistance to three or more antibiotic classes, was observed in four (1.9%) isolates.

**Figure 2:**
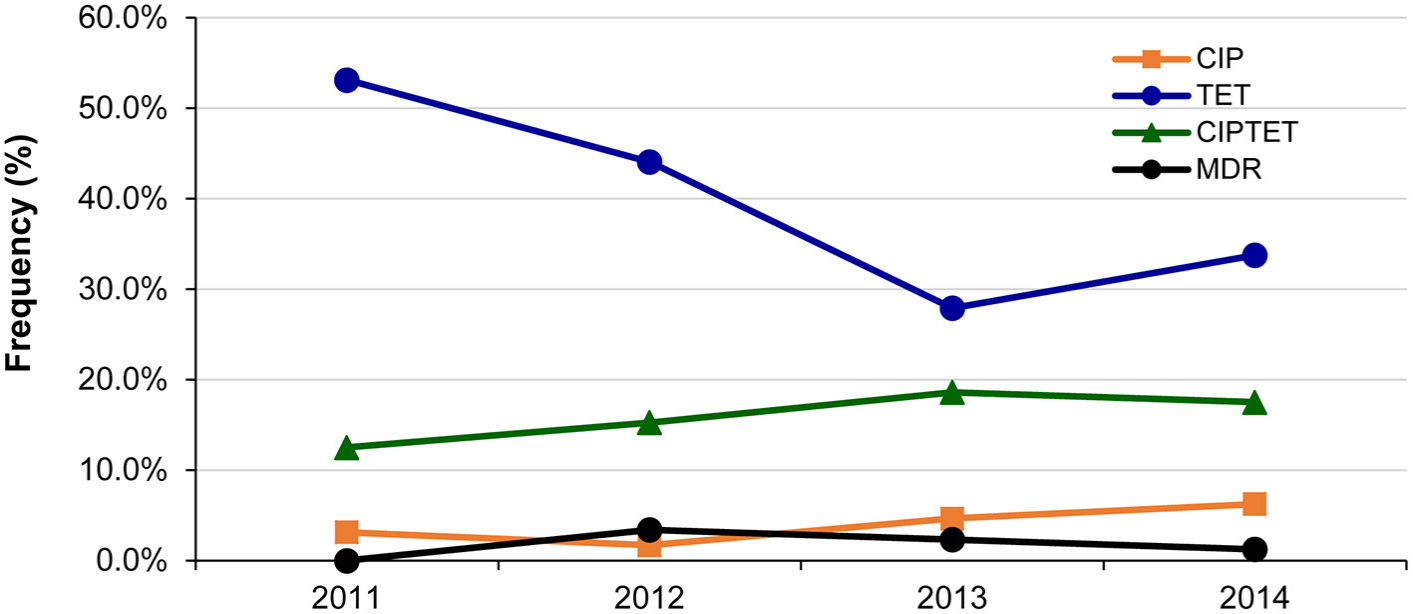
Changes in the frequency of the most common antibiotic resistance phenotypes among 214 *Campylobacter jejuni* isolates over a 4-year period in Michigan. CIP, ciprofloxacin; TET, tetracycline. Multidrug resistant (MDR) isolates were resistant to CIPAZIERYCLITEL, CIPTETCLI, and CIPTETTEL.

Fluctuations in resistance frequencies were observed by year. Although no significant increase in any resistance or MDR was observed over the 4-year period, notable trends were observed for some phenotypes (Figure 2). For instance, a significant decrease in the frequency of isolates with only tetracycline resistance was observed over time (*p* = 0.04), while a slight insignificant increase in ciprofloxacin resistance was observed alone (*p* ≤ 0.24) and in combination with tetracycline resistance (*p* ≤ 0.50). Despite the gradual increase in the frequency of any resistance to ciprofloxacin from 15.6% in 2011 to 25.0% in 2014, the change was not significant (*p* ≤ 0.31). The same was true for any resistance to tetracycline, which decreased from 65.6% in 2011 to 52.5% in 2014 (*p* = 0.17).

### Epidemiological Associations with Antibiotic Resistant *C. jejuni* Infections

Several notable associations were identified between epidemiological factors and the most common antibiotic resistant phenotypes, ciprofloxacin resistance and tetracycline resistance. These two predominant phenotypes were classified as the dependent variables to uncover associations with each phenotype relative to cases with either susceptible infections or infections with resistance to all other antibiotics.

Patients with ciprofloxacin resistance (*n* = 49) were more likely to travel in the month prior to infection (OR: 3.0; 95% CI: 1.37, 6.68) relative to all other cases (Table 1). More specifically, they were more likely to report international travel (OR: 9.8; 95% CI: 3.69, 26.09). Patients with tetracycline resistance (*n* = 120) were also more likely to report international travel in the past month, however, the difference was not significant (OR: 2.2; 95% CI: 0.85, 5.46). Patients with tetracycline resistance were also significantly less likely to have an infection during the summer or fall months (OR: 0.5; 95% CI: 0.27, 0.97) and to report contact with livestock (Fisher's exact test *p* = 0.04) or well water (OR: 2.3; 95% CI: 0.95, 5.75); the latter association was not significant. No association was observed between resistance to either antibiotic and domestic travel history, hospitalization, or clinical symptoms including body aches, diarrhea with blood, fatigue, fever, abdominal pain, and headache.

**Table 1:** Univariate analysis to identify factors associated with any ciprofloxacin resistance (CIP) and any tetracycline resistance (TET) among 214 *Campylobacter jejuni* isolates from Michigan, 2011 to 2014.

	**Any CIP resistance (*n =* 49)**				**Any TET resistance (*n =* 120)**			
**Characteristics^a^**	**No**.	**(%)**	**OR (95% CI)^b^**	***p*-value^c^**	**No**.	**(%)**	**OR (95% CI)^b^**	***p*-value^c^**
**Age (years)**								
0–9 (*n =* 65)	14	(21.5)	0.7 (0.31, 1.65)	0.43	39	(60.0)	1.1 (0.53, 2.32)	0.77
10–18 (*n =* 18)	2	(11.1)	–	0.21	6	(33.3)	0.4 (0.12, 1.14)	0.08
19–40 (*n =* 54)	15	(27.8)	1.0	–	31	(57.4)	1.0	–
41–65 (*n =* 59)	16	(27.1)	1.0 (0.42, 2.21)	0.94	33	(55.9)	0.9 (0.45, 1.98)	0.87
≥65 (*n =* 17)	2	(11.8)	-	0.21	10	(58.8)	1.1 (0.35, 3.20)	0.92
**Sex**								
Male (*n =* 110)	23	(20.9)	1.0	–	58	(52.7)	1.0	–
Female (*n =* 96)	23	(24.0)	0.8 (0.44, 1.62)	0.60	54	(56.3)	1.2 (0.66, 2.00)	0.61
**Self-reported race^d^**								
White/Caucasian (*n =* 137)	32	(23.4)	1.0	–	76	(36.5)	1.0	–
Non-white/other (*n =* 35)	9	(25.7)	1.1 (0.48, 2.67)	0.77	17	(48.6)	0.8 (0.36, 1.59)	0.46
**Arab ethnicity**								
No (*n =* 106)	27	(25.5)	–	–	53	(50.0)	1.0	–
Yes (*n =* 25)	3	(12.0)	–	0.19	17	(68.0)	2.1 (0.84, 5.35)	0.10
**Season**								
Winter, Spring (*n =* 56)	12	(21.4)	1.0	–	38	(67.9)	1.0	–
Summer, fall (*n =* 158)	37	(23.4)	1.1 (0.54, 2.34)	0.76	82	(51.9)	0.5 (0.27, 0.97)	0.04
**Any travel in the past month**								
No (*n =* 88)	13	(14.8)	1.0	–	45	(51.1)	1.0	–
Yes (*n =* 61)	21	(34.4)	3.0 (1.37, 6.68)	0.005	37	(60.7)	1.5 (0.76, 2.86)	0.25
**Type of travel in the past month**								
None (*n =* 88)	13	(14.8)	1.0	–	45	(51.1)	1.0	–
Domestic (*n =* 33)	4	(12.5)	–	1.0	18	(56.3)	1.1 (0.51, 2.56)	0.74
International (*n =* 27)	17	(63.0)	9.8 (3.69, 26.09)	<0.0001	18	(69.2)	2.2 (0.85, 5.46)	0.10
**Type of drinking water**								
Municipal, bottled (*n =* 119)	25	(21.1)	1.0	–	60	(50.4)	1.0	–
Any well water (*n =* 27)	5	(18.5)	0.9 (0.29, 2.48)	0.77	19	(70.4)	2.3 (0.95, 5.75)	0.06
**Poultry consumption**								
No (*n =* 16)	4	(25.0)	–	–	10	(62.5)	1.0	
Yes (*n =* 115)	24	(20.9)	–	0.75	61	(53.0)	0.7 (0.23, 1.99)	0.48
**Any animal contact**								
No (*n =* 54)	12	(22.2)	1.0	–	30	(55.6)	1.0	-
Yes (*n =* 96)	20	(20.8)	0.9 (0.41, 2.07)	0.84	52	(54.2)	0.9 (0. 48, 1.85)	0.87
**Contact with livestock**								
No (*n =* 137)	31	(22.6)	–	–	71	(51.8)	–	–
Yes (*n =* 13)	1	(7.7)	–	0.30	11	(84.6)	–	0.04
**Cattle density in resident county^e^**								
Low <8,400 cattle (*n =* 23)	3	(13.0)	–	–	12	(52.2)	1.0	–
High ≥8,400 cattle (*n =* 82)	21	(25.6)	–	0.27	50	(61.0)	1.4 (0.56, 3.63)	0.45
**Residence type**								
Rural (*n =* 73)	18	(24.7)	1.0	–	45	(61.6)	1.0	–
Urban (*n =* 119)	25	(21.0)	0.8 (0.41, 1.62)	0.56	62	(52.1)	0.7 (0.37, 1.22)	0.20
**Hospitalized**								
No (*n =* 136)	27	(19.9)	1.0		80	(58.8)	1.0	–
Yes (*n =* 46)	14	(30.4)	1.8 (0.83, 3.76)	0.14	21	(45.7)	0.6 (0.30, 1.15)	0.12

a *Not all numbers add up to the total number of cases per category due to missing data for some variables or the exclusion of susceptible isolates*.

b *The 95% confidence interval (CI) for the odds ratio (OR) is presented; ORs were calculated separately for CIP and TET relative to all other isolates*.

c *The Fisher's Exact Test was used for variables with ≤ 5 in one cell; no ORs could be calculated*.

d *Self-reported race categories in the online Michigan Disease Surveillance System questionnaire were: Caucasian, African American, Asian, American Indian/Alaska Native, Hawaiian/Pacific Islander, Unknown, or Other*.

e *Cattle density was not known for multiple counties with high case counts*.

Because a subset of the isolates had both tetracycline and ciprofloxacin resistance, we created mutually exclusive categories to identify risk factors for each. In this analysis, ciprofloxacin resistance was defined as any resistance to ciprofloxacin even if resistance to other drugs including tetracycline was observed. Among the 135 resistant isolates, 49 (36.3%) had ciprofloxacin resistance. Tetracycline resistance was defined as any resistance to tetracycline but without the co-occurrence of ciprofloxacin resistance; 83 (61.5%) isolates had tetracycline resistance without ciprofloxacin resistance. Individuals with susceptible isolates and those representing different resistance profiles were excluded from the analysis. Compared to patients with tetracycline resistance, those with ciprofloxacin resistant infections were significantly more likely to report traveling in the past month (OR: 2.9; 95% CI: 1.20, 7.02) and specifically, international travel (Fisher's exact test *p* < 0.0001) (Table 2). Only four (19.1%) of the 21 patients who traveled internationally had tetracycline resistant infections compared to 17 (81.0%) of those with ciprofloxacin resistant infections. A difference was also observed for hospitalization, which was significantly more common in ciprofloxacin resistant infections (OR: 2.5, 95% CI: 1.02, 6.14), while contact with livestock was more common in tetracycline resistant infections (Fisher's exact test *p* = 0.09), yet the latter association was not significant. No association was observed for age, sex, race, ethnicity, residence types, season, water source, cattle density, poultry consumption, or clinical symptoms.

**Table 2:** Epidemiological factors associated with any ciprofloxacin resistance (CIP) vs. only tetracycline resistance (TET) among 135 patients with resistant infections.

	**Any CIP resistance (*n =* 49)**		**Only TET resistance (*n =* 83)**			
**Characteristics^a^**	**No**.	**(%)**	**No**.	**(%)**	**OR (95% CI)^b^**	***p*-value^c^**
**Age (years)**						
0–40 (*n =* 83)	31	(37.4)	52	(62.5)	1.0	–
≥41 (*n =* 48)	18	(37.5)	30	(62.5)	1.0 (0.48, 2.10)	0.99
**Sex**						
Male (*n =* 65)	23	(35.4)	42	(64.6)	1.0	–
Female (*n =* 59)	23	(39.0)	36	(61.0)	1.2 (0.41, 1.78)	0.85
**Residence type**						
Rural (*n =* 48)	18	(37.5)	30	(37.5)	1.0	–
Urban (*n =* 70)	25	(35.7)	45	(64.3)	0.9 (0.43, 1.98)	0.84
**Season**						
Winter, spring (*n =* 39)	12	(30.8)	27	(69.2)	1.0	–
Summer, fall (*n =* 93)	37	(39.8)	56	(60.2)	1.5 (0.67, 3.30)	0.33
**Any travel in the past month**						
No (*n =* 49)	13	(26.5)	36	(73.5)	1.0	–
Yes (*n =* 41)	21	(51.2)	20	(48.8)	2.9 (1.20, 7.02)	0.02
**Type of travel in the past month**						
None (*n =* 49)	13	(26.5)	36	(73.5)	1.0	–
Domestic (*n =* 19)	4	(21.1)	15	(79.0)	–	0.76
International (*n =* 21)	17	(81.0)	4	(19.1)	–	<0.0001
**Type of drinking water**						
Municipal, bottled (*n =* 67)	25	(37.3)	42	(62.7)	1.0	–
Any well water (*n =* 20)	5	(25.0)	15	(75.0)	–	0.42
**Poultry consumption**						
No (*n =* 16)	4	(40.0)	6	(60.0)	–	–
Yes (*n =* 115)	24	(35.3)	44	(64.7)	–	0.77
**Contact with livestock**						
No (*n =* 79)	31	(39.2)	48	(60.8)	–	–
Yes (*n =* 11)	1	(9.1)	10	(90.9)	–	0.09
**Hospitalized**						
No (*n =* 85)	27	(31.8)	58	(68.2)	1.0	–
Yes (*n =* 26)	14	(53.9)	12	(46.2)	2.5 (1.02, 6.14)	0.04
Multivariate analysis^d^					Adjusted OR (95% CI)	*p*-value
Age					1.0 (0.98, 1.02)	0.87
Female					0.5 (0.17, 1.40)	0.18
Urban residence					1.0 (0.33, 2.83)	0.95
Summer or fall infection					3.7 (1.03, 13.47)	0.04
International travel only					14.9 (4.00, 55.57)	<0.0001
Hospitalized					3.0 (0.78, 11.19)	0.11
Well water					0.6 (0.16, 2.26)	0.44
Livestock contact					0.2 (0.02, 2.25)	0.21

a *Number of isolates may not add up to the total for some variables due to missing data; percentages were calculated using the number with each characteristic as the denominator*.

b *95% confidence interval for the odds ratio (OR). ORs were calculated for ciprofloxacin resistance relative to tetracycline resistance*.

c *The Fisher's Exact test was used for variables with fewer than 5 in one cell; no ORs could be calculated*.

d *Multivariate results were generated using forward stepwise logistic regression while controlling for variables with p-values ≤ 0.2 in the univariate analysis as well as potential confounders. A base model consisted of the following variables: age (continuous), female sex, urban residence, season (fall and summer), and international travel. Each additional variable was added separately to the base model. The Homer and Lemeshow Goodness-of-Fit test (p > 0.05) was examined to ensure support for each model. Adjusted ORs were calculated and the Wald Chi-Square test was used to determine significance with 95% Wald Confidence Limits*.

Multinomial logistic regression was performed to identify predictors of ciprofloxacin resistance relative to tetracycline resistance while controlling for age, sex, urban residence, season, and international travel. Notably, international travel in the past month (adjusted OR: 13.0; 95% CI: 3.71, 45.64) and infection during the summer or fall months were the only significant predictors of ciprofloxacin resistance (Table 2). Hospitalization was also more common in patients with ciprofloxacin resistance than tetracycline resistance, yet the association was not significant in the model (adjusted OR: 2.4; 95% CI: 0.86, 6.46), which could be due to the small sample size.

### Comparing Resistance Frequencies to National Data Reported *Via* NARMS

**Figure 3:**
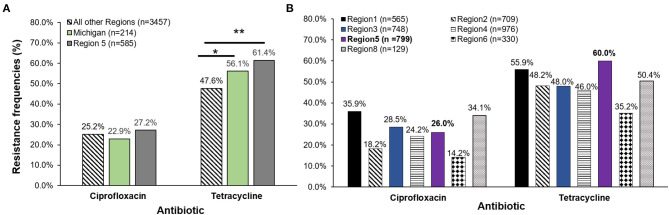
**(A)** Antibiotic resistance frequencies of *Campylobacter jejuni* strains recovered from four Michigan hospitals (*n* = 214) in 2011–2014 as compared to the National Antimicrobial Resistance Monitoring System (NARMS) data for the same time period. Michigan frequencies were compared to NARMS data from Region 5 acquired from Minnesota (representing Ohio, Indiana, Michigan, Illinois, Wisconsin, and Minnesota and 34 federally recognized tribes) and the total national data (excluding Region 5). **(B)** Michigan frequencies were added to Region 5 national data (*n* = 585) leaving a total of 799 strains in the Midwest region for comparison to NARMS regions 1, 2, 3, 4, 6, and 8. *p ≤ 0.05, **p ≤ 0.0001; χ^2^ test. The 10 FoodNet sites representing Connecticut, Georgia, Maryland, Minnesota, New Mexico, Oregon, Tennessee, California, Colorado, and New York send data captured by the state public health laboratories to NARMS to represent the different regions. Data from Region 1 (Connecticut, Maine, Massachusetts, New Hampshire, Rhode Island, and Vermont), Region 2 (New Jersey, New York, Puerto Rico, and the Virgin Islands), Region 3 (Delaware, District of Columbia, Maryland, Pennsylvania, Virginia, and West Virginia), Region 4 (Alabama, Florida, Georgia, Kentucky, Mississippi, North Carolina, South Carolina, and Tennessee), Region 6 (Arkansas, Louisiana, New Mexico, Oklahoma, and Texas), and Region 8 (Colorado, Montana, North Dakota, South Dakota, Utah and Wyoming) were included in the analysis. Regions 7, 9, and 10 did not have data available for *Campylobacter jejuni* from 2011 to 2014 for comparison.

A comparison of resistance frequencies for the NARMS isolates recovered during the same time period uncovered region-specific differences for both ciprofloxacin and tetracycline resistance. A significantly greater proportion of the Michigan isolates were resistant to tetracycline when compared to the 3,457 isolates from all regions except Region 5. Although Region 5 covers Michigan and other midwestern states, the data were generated by examining only a subset of isolates recovered from the Minnesota FoodNet site (Figure 3A). No difference in tetracycline resistance frequencies was observed between Michigan and the Region 5 isolates (*n* = 585), or for any of the ciprofloxacin resistance frequencies. However, when our Michigan isolates (*n* = 214) were added to Region 5, differences were observed for both ciprofloxacin and tetracycline resistance across the NARMS regions (Figure 3B). Notably, Region 5 had significantly more tetracycline resistance than all other regions combined (OR: 1.6; 95% CI: 1.41, 1.92) as well as a significantly greater proportion of ciprofloxacin resistance than Regions 2 (OR: 1.6; 95% CI: 1.24, 2.03) and 6 (OR: 2.1; 95% CI: 1.50, 3.00). Relative to Region 1, however, the proportion of ciprofloxacin resistance was significantly lower than in Region 5 (OR: 0.6; 95% CI: 0.50, 0.79). No other differences were observed for ciprofloxacin resistance by region.

## Discussion

Through this study we have detected important trends in the prevalence of campylobacteriosis and antibiotic resistant *C. jejuni* isolated from Michigan patients between 2011 and 2014, further highlighting the importance of pathogen surveillance efforts using culture-based methods. Since campylobacteriosis was not classified as a notifiable infection until 2015 (25), data about disease frequencies and resistance profiles have been limited, particularly for states like Michigan that are not participating in FoodNet or NARMS. In addition, the widespread adoption of culture-independent tests has hampered the ability to routinely monitor important phenotypes such as antibiotic susceptibility profiles. Indeed, it was estimated that 42% of campylobacteriosis cases identified via FoodNet in 2019 were diagnosed by culture-independent tests and among these, culture for *Campylobacter* was only attempted for 63% of the positive samples (2).

In the four Michigan hospitals examined herein, we observed a significant increase in *C. jejuni* infections over time, which is similar to national trends (2) and could partly be due to improved sampling and detection capacity. Seasonal differences were also observed with a greater proportion (73.8%) of Michigan cases occurring during the summer and fall. Seasonal variation has been reported previously with several studies showing a peak incidence of *C. jejuni* infections during warmer months; climate, temperature, increased shedding from animal reservoirs, and/or seasonal-specific behaviors have all been suggested to contribute to seasonality (26–28).

Extracting epidemiological data from case records has also facilitated the identification of factors that increase risk of campylobacteriosis. Similar to our prior study of 7,182 campylobacteriosis cases reported in Michigan between 2004 and 2013 (29) and those from the FoodNet sites (25, 30), most infections affected children <10 (30.8%) or adults between 19 and 65 (53.1%) years of age. Despite this bimodal distribution, the likelihood of hospitalization increased with increasing age. Cases between 41 and 65 years were significantly more likely to be hospitalized than those between 19 and 40 years of age as were cases over 65. The link between older age and more severe disease has been reported for the FoodNet sites and in our prior population-based study (29, 31).

Although males and rural residents represented a greater proportion of the 7,182 campylobacteriosis cases in Michigan (29), similar distributions were not observed among the cases at the four hospitals. For instance, no difference was observed by sex and significantly more cases (62.0%) were from urban areas, suggesting that the four hospitals may not be entirely representative of the Michigan population of campylobacteriosis cases. Such differences are likely due to the structure of the surveillance system since we utilized four of the largest health care systems. Despite having wide catchment areas, each hospital is in a metropolitan location that can result in differences in access to health care, particularly for rural residents, supporting the suggestion that geography as well as patient-specific and cultural factors can impact care seeking behaviors (11). Indeed, we observed a lower likelihood of hospitalization among rural residents in this and our prior study (29), although this association was not significant after controlling for race, sex, and age.

Because the likelihood of hospitalization was significantly greater for cases self-identifying as non-White, urban areas should be an important focus for reducing disparities in infections caused by *C. jejuni* and other enteric pathogens. Certainly, neighborhood and geographic barriers have previously been suggested to be important for the acquisition of foodborne disease (13). Although race, ethnicity, and other socially constructed categorizations such as socio-economic status, are not typically collected for foodborne disease surveillance systems, prior studies have document increased frequencies of gastroenteritis in minority and low-socioeconomic populations globally (32–34). Additional studies are needed, however, to identify specific risk factors, exposures and causal factors within urban environments that may explain these relationships. Use of previously reported proxies and markers of poverty such as urban residence, and social constructs like self-reported race as we have used, have complex interactions with social determinants of health (35, 36). We therefore cannot describe causal factors for hospitalization of *C. jejuni* without addressing these shortcomings. We also cannot rule out the possibility that different strain populations with distinct pathogenic traits are circulating in the different areas and are partly responsible for the differences observed.

Since most hospital laboratories in Michigan have switched to the use of culture-independent tests to detect *C. jejuni*, viable isolates are not typically recovered for characterizing important phenotypic or genotypic traits. Hence, our assessment of resistance frequencies and trends in the four hospitals over this 4-year period yielded notable results. Resistance was detected in 63.1% of the 214 isolates and to all nine antibiotics comprising 11 distinct resistance profiles. The overall predominance of tetracycline (56.1%) and ciprofloxacin (22.9%) resistance was similar in our prior study of 94 isolates recovered in 2011 and 2012 (22). The inclusion of 120 additional isolates recovered from the same hospitals in 2013–2014, however, allowed for the detection of several important changes over time, including an increase in the frequency of fluoroquinolone resistance. This gradual increase is concerning given that fluoroquinolones are commonly used to treat human infections and the Food and Drug Administration (FDA) banned use of these drugs in poultry in 2005 (37). Point mutations in chromosomal genes such as *gyrA*, which is critical for DNA replication and transcription, have been linked to fluoroquinolone resistance (38, 39). Given that these mutations do not halt transcription, there is no impact on bacterial survival and hence, these resistant bacterial populations can persist in the absence of antibiotic selection (40, 41). This increasing frequency of ciprofloxacin resistance in *C. jejuni* is consistent with national trends for older strain sets recovered via culture-based detection methods (25). Because of the increased use of culture-independent methods to detect *Campylobacter*, however, actual rates of ciprofloxacin resistance among clinical isolates in different parts of the U.S. are not well-established. Additionally, despite the critical role that FoodNet and NARMS have played in the detection of resistant foodborne pathogens, neither system is entirely representative of the U.S. population (12). To represent the entire Midwest (Region 5) that includes Michigan, for instance, NARMS only receives a subset of isolates from Minnesota for testing (18). It is therefore important to note that the frequencies and trends reported by NARMS may not accurately reflect those observed in other locations with distinct geographic features and population traits.

Furthermore, fluoroquinolone resistant *C. jejuni* infections have also been reported to increase the duration of illness (42, 43). Mutations in *gyrA* have been tied to changes in DNA supercoiling, which can lead to enhanced colonization of the chicken gut and an increase in virulence properties such as motility, biofilm formation and invasion of intestinal epithelial cells *in vitro* (44–47). These studies establish a mechanism by which fluoroquinolone resistant mutations enhance virulence and support prior associations between resistance and a lengthier illness duration. Additional support comes from our finding that patients with ciprofloxacin resistant infections were twice as likely to be hospitalized than patients with tetracycline resistant infections, suggesting that the former may be more severe. It is not clear, however, if a unique patient population, differential treatment regimens, or distinct bacterial factors account for the difference in the hospitalization rates observed.

Significant differences in the frequency of tetracycline resistance, which was highest in this population of Michigan patients despite the gradual decrease in tetracycline resistance over time, were also observed. These data indicate that unique regional factors may impact resistance rates, yet those factors that contribute to variation across locations are not clear. The tetracyclines have been used to treat zoonotic and rickettsial diseases in human medicine (48) and have been used extensively in livestock and poultry production worldwide. In the U.S., the FDA reported that tetracyclines were the predominant drug class used in food-producing animals at the time of this study (2009–2014), representing an average of 42% of all antibiotics used (49). Continuous use of tetracycline has selected for resistant strains and resistance genes that can persist in reservoir hosts and the environment. For example, TetO has been shown to mediate resistance to tetracycline in *C. jejuni* by offering ribosomal protection by binding to an unoccupied site (39). This protein is encoded by *tet*(O), which is commonly carried on the pTet plasmid but has also been detected in the chromosome (50, 51). Given the high transmissibility rates of these resistance plasmids within bacterial populations even in the absence of tetracycline use (52, 53), it is clear that *C. jejuni* serves as an important reservoir for these and other resistance genes. In our prior study, we demonstrated that tetracycline resistance was more common in strains belonging to multilocus sequence type (ST)-982, a lineage that was also common in Michigan cattle (22, 54) and has been linked to livestock in other locations (55, 56). Together, these data show the importance of clonal expansion of resistant lineages and highlight the role that mobile genetic elements play in dispersion and maintenance of tetracycline resistance.

Although we observed a significant association between livestock contact and tetracycline resistance, the number of cases (*n* = 13) reporting this exposure was low. It is noteworthy, however, that only one of the cases reporting contact with livestock had a ciprofloxacin resistant infection compared to 11 (84.6%) with tetracycline resistant infections. While increasing frequencies of ciprofloxacin resistant *C. jejuni* have been recovered from feedlot cattle throughout the U.S. (57), our data suggest that different factors are important for the acquisition of ciprofloxacin vs. tetracycline resistant infections. Consistent with prior studies (25, 30, 42, 58), we have demonstrated that international travel in the month prior to infection is the strongest predictor of ciprofloxacin resistance in this sample of Michigan patients. Infection during the summer or fall months was also independently associated with ciprofloxacin resistance, but we did not observe an association with poultry consumption as was described in other studies (42, 58, 59). This difference could be due to the low number of cases reporting no poultry consumption a week before symptom onset or the high frequency of missing data since many patients failed to answer the food history questions, a common problem with long-term epidemiological studies (60). In general, however, the identification of risk factors that have also been described in other studies is encouraging and indicates that these factors are likely important regardless of the geographic location.

Collectively, the data presented herein demonstrate the importance of monitoring antibiotic resistance phenotypes and frequencies using culture-based methods in multiple geographic locations. The significant difference that we observed in NARMS Region 5 relative to other regions after including our Michigan data with those from Minnesota, illustrates the need for more comprehensive testing and highlights the variation across different geographic locations. Future studies are still needed, however, to link resistance profiles and patient data to epidemiological data to identify those exposures and risk factors that are unique to specific states or regions.

## Data Availability Statement

The original contributions presented in the study are included in the article/Supplementary Material, further inquiries can be directed to the corresponding author/s.

## Ethics Statement

The studies involving human participants were reviewed and approved by Institutional Review Boards at Michigan State University (10-735SM), the Michigan Dept. of Health and Human Services (842-PHALAB) and each of the four participating hospitals. Written informed consent from the participants' legal guardian/next of kin was not required to participate in this study in accordance with the national legislation and the institutional requirements.

## Author Contributions

SDM, WC, and JAR designed the study. JTR, DWN, HS, PL, and WK organized sample collection at each site. REM and WC isolated pathogens and extracted epidemiological data. JAR and WC performed the experiments. JAR, SM, and SDM managed the data and conducted analyses. JAR developed the first manuscript draft. All authors contributed and approved the manuscript content.

## Conflict of Interest

The authors declare that the research was conducted in the absence of any commercial or financial relationships that could be construed as a potential conflict of interest.
