# Supplementary material for: Epidemiologic Associations Vary Between Tetracycline and Fluoroquinolone Resistant Campylobacter jejuni Infections
Source: Front Public Health. 2021 Jun 28;9:672473. doi: 10.3389/fpubh.2021.672473 (PMC8273344; doi:10.3389/fpubh.2021.672473)
Supplement: Supplementary file 1 [file Data_Sheet_1.PDF]

Table S1. Differences in the proportion of specific characteristics among 214 cases infected with *Campylobacter jejuni* in Michigan, 2011-2014.

Case characteristics	Number [a]	(%)	Chi square [b]	DF	p value
Age (years)			50.7	4	<0.0001
0-9	65	(30.5)			
10-18	18	(8.5)			
19-40	54	(25.4)			
41-65	59	(27.7)			
>65	17	(8.0)			
Sex			0.95	1	0.33
Male	110	(53.4)			
Female	96	(46.6)			
Self-reported race [c]			275.4	3	<0.0001
White/Caucasian	137	(79.7)			
Black/African American	17	(9.9)			
Asian	6	(3.5)			
Other	12	(7.0)			
Self-reported Hispanic ethnicity			99.6	1	<0.0001
Non-Hispanic/Latino	134	(91.2)			
Hispanic/Latino	13	(8.8)			
Self-reported Arab ethnicity			50.1	1	<0.0001
Non-Arab	106	(80.9)			
Arab	25	(19.1)			
County classification			11.0	1	0.0009
Rural	73	(38.0)			
Urban	119	(62.0)			
Cattle density in resident county [d]			33.1	1	<0.0001
Low (<8400 cattle)	23	(21.9)			
High (≥8400 cattle)	82	(78.1)			
Any travel in the past month			4.9	1	0.03
No	88	(59.1)			
Yes	61	(40.9)			
Type of travel in the past month			46.8	2	<0.0001
None	88	(59.9)			
Domestic travel only	33	(22.4)			
Any international travel	27	(18.4)			

Animal contact			11.8	1	0.0006
No	54	(36.0)			
Yes	96	(64.0)			
Poultry consumption in the past week			74.8	1	<0.0001
No	16	(12.2)			
Yes	115	(87.8%)			
Drinking water			58.0	1	<0.0001
Municipal, bottled	119	(81.5)			
Any well water	27	(18.5)			

[a] Not all numbers add up to the total number of 214 cases due to missing data for some variables.

[b] Significant differences were identified using the Chi-square Test for Equal Proportions; DF = degree of freedom.

[c] Self-reported race categories in the online Michigan Disease Surveillance System questionnaire were: Caucasian, African American, Asian, American Indian/Alaska Native, Hawaiian/Pacific Islander, Unknown, or Other.

[d] Cattle density was not known for multiple counties with high case counts.

Table S2. Demographic, epidemiologic, and clinical characteristics associated with urban versus rural residence

Characteristics	Urban residents (n=119) No. (%)	Rural residents (n=73) No. (%)	OR (95% CI) [b]	p value [c]
Age (years)				
0-9	46 (83.6)	9 (16.4)	9.3 (3.69, 23.56)	<0.0001
10-18	14 (77.8)	4 (22.2)	-	0.003
19-40	17 (35.4)	31 (64.6)	1.0	-
41-65	34 (60.7)	22 (39.3)	2.8 (1.27, 6.26)	0.01
≥65	8 (53.3)	7 (46.7)	2.1 (0.62, 6.97)	0.22
Sex				
Male	62 (62.0)	38 (38.0)	1.0	-
Female	54 (60.7)	35 (39.3)	0.9 (0.53, 1.70)	0.85
Self-reported race [d]				
White/Caucasian	82 (78.1)	54 (81.8)	1.0	-
Non-White/Other	23 (21.9)	12 (18.2)	1.3 (0.58, 2.75)	0.70
Hispanic ethnicity				
No	84 (63.2)	49 (36.8)	-	-
Yes	8 (61.4)	5 (38.5)	-	1.0
Arab ethnicity				
No	60 (57.1)	45 (42.9)	-	-
Yes	24 (96.0)	1 (4.0)	-	0.0001
Any travel in the past month				
No	59 (67.1)	29 (33.0)	1.0	0.006
Yes	27 (44.3)	34 (55.7)	0.4 (0.20, 0.77)	
Type of travel in the past month				
None	59 (67.1)	29 (33.0)	1.0	-
Domestic	14 (42.4)	19 (57.6)	0.4 (0.20, 0.80)	0.01
International	14 (51.9)	13 (48.2)	0.5 (0.22, 1.27)	0.15
Drinking water				
Municipal, bottled	73 (61.3)	46 (38.7)	1.0	-
Any well water	12 (44.4)	15 (55.6)	0.5 (0.22, 1.17)	0.11
Exposure to livestock				
No	83 (60.6)	54 (39.4)	-	-
Yes	3 (23.1)	10 (76.9)	-	0.02

Hospitalized				
No	77 (56.6)	59 (43.4)	1.0	-
Yes	34 (73.9)	12 (26.1)	2.2 (1.04, 4.55)	0.04

[a] Number of isolates may not add up to the total for some variables due to missing data. For each category, percentages were calculated using the number with each characteristic as the denominator

[b] 95% confidence interval for the odds ratio (OR). ORs were calculated for urban residence relative to rural residence.

[c] The Fisher's Exact test was used for variables with fewer than 5 in one cell; no ORs could be calculated

[d] Self-reported race categories in the online Michigan Disease Surveillance System questionnaire were: Caucasian, African American, Asian, American Indian/Alaska Native, Hawaiian/Pacific Islander, Unknown, or Other.

Table S3: Univariate and multivariate analysis identifying predictors of hospitalization among 214 patients with *Campylobacter jejuni* infections.

Characteristics	No. of Cases [a]	Hospitalized No.	(%)	OR (95% CI) [b]	p value [c]
**Demographics**					
Age (years)					
0-9	50	6	(12.0)	-	1.0
10-18	17	5	(29.4)	-	0.12
19-40	44	5	(11.4)	1.0	-
41-65	56	23	(41.1)	-	0.001
≥65	15	7	(46.7)	-	0.007
Sex					
Male	95	24	(25.3)	1.0	-
Female	84	22	(26.2)	1.0 (0.54, 2.01)	0.89
Self-reported race					
White/Caucasian	130	29	(22.3)	1.0	0.01
Non-White/Other [d]	34	15	(44.1)	2.7 (1.24, 6.08)	
Hispanic ethnicity					
No	131	41	(31.3)	-	0.18
Yes	12	1	(8.3)	-	
Arab ethnicity					
No	104	39	(37.5)	-	0.007
Yes	23	2	(8.7)	-	
Residence by county					
Rural	71	12	(16.9)	1.0	-
Urban	111	34	(30.6)	2.2 (1.04, 4.55)	0.04
**Exposure history**					
Season infected					
Winter, Spring	49	9	(18.4)	1.0	-
Summer, Fall	133	37	(27.8)	1.7 (0.76, 3.88)	0.19
Any travel in the past month					
No	87	32	(36.8)	1.0	0.007
Yes	61	10	(16.4)	0.3 (0.15, 0.75)	
Travel in the past month					
None	87	32	(36.8)	1.0	-
Domestic travel only	33	6	(18.2)	0.4 (0.14, 1.02)	0.05
International travel only	27	4	(14.8)	-	0.03

Drinking water					
Municipal, bottled	118	36	(30.5)	-	0.25
Any well water	27	5	(18.5)	-	
**<u>Clinical symptoms</u>**					
Diarrhea with blood					
No	108	33	(30.6)	1.0	-
Yes	63	13	(20.6)	0.6 (0.28, 1.23)	0.16
Abdominal pain					
No	50	16	(32.0)	1.0	-
Yes	123	30	(24.4)	0.7 (0.33, 1.41)	0.30
Body aches					
No	122	30	(24.6)	1.0	-
Yes	51	16	(31.4)	1.4 (0.68, 2.88)	0.36
Fatigue					
No	99	21	(21.2)	1.0	-
Yes	72	25	(34.7)	2.0 (1.00, 3.91)	0.05
Nausea					
No	99	18	(18.2)	1.0	-
Yes	73	28	(38.4)	2.8 (1.40, 5.61)	0.003

**Multivariate analysis** [e]	**Adjusted OR (95% CI)**	**p value**
Age 41 to 65 years	6.1 (2.37, 15.70)	0.003
Age ≥65 years	10.5 (2.63, 42.19)	0.003
Non-White/Other race [d]	4.8 (1.62, 14.01)	0.009
International travel	0.3 (0.07, 0.94)	0.04
Nausea	2.8 (1.15, 6.68)	0.03

[a] Number of isolates may not add up to the total for some variables due to missing data.

[b] 95% confidence interval for the odds ratio (OR)

[c] The Fisher's Exact Test was used for variables with fewer than 5 in one cell; no ORs could be calculated

[d] Other (Non-White) race includes the following self-reported categories: Asian, Black/African American, and Other.

[e] Multivariate results were generated using forward stepwise logistic regression while controlling for variables with p-values ≤0.2 in the univariate analysis as well as potential confounders. The variables were: age, sex, residence location (urban versus rural), season (fall and summer versus spring and winter), race (Non-White versus White/Caucasian), international travel, domestic travel, and symptoms (fatigue and nausea). The Homer and Lemeshow Goodness-of-Fit test indicates that the model is supported (Chi-Square=5.36; degrees of freedom=7; p=0.62) despite using only 133 of the 214 case records. Adjusted ORs were calculated and the Wald Chi-Square test was used to determine significance with 95% Wald Confidence Limits.
